# Expression of concern: An efficient and green procedure for synthesis of rhodanine derivatives by aldol-thia-Michael protocol using aqueous diethylamine medium

**DOI:** 10.1039/d5ra90007g

**Published:** 2025-01-15

**Authors:** Assem Barakat, Abdullah M. Al-Majid, Hany J. AL-Najjar, Yahia N. Mabkhot, Hazem A. Ghabbour, Hoong-Kun Fun

**Affiliations:** a Department of Chemistry, College of Science, King Saud University P. O. Box 2455 Riyadh 11451 Saudi Arabia ambarakat@ksu.edu.sa +966 1-4675992 +966 1-4675884; b Department of Chemistry, Faculty of Science, Alexandria University P.O. Box 426, Ibrahimia 21321 Alexandria Egypt; c Department of Pharmaceutical Chemistry, College of Pharmacy, King Saud University P. O. Box 2457 Riyadh 11451 Saudi Arabia

## Abstract

Expression of concern for ‘An efficient and green procedure for synthesis of rhodanine derivatives by aldol-thia-Michael protocol using aqueous diethylamine medium’ by Assem Barakat *et al.*, *RSC Adv.*, 2014, **4**, 4909–4916, https://doi.org/10.1039/C3RA46551A.

*RSC Advances* is publishing this expression of concern in order to alert readers about concerns with the integrity of the NMR data in the supplementary information. An expression of concern will continue to be associated with the article until a conclusive outcome is reached.

Laura Fisher

3rd January 2025

Executive Editor, *RSC Advances*

